# Quantifying the influence of the tobacco industry on EU governance: automated content analysis of the EU Tobacco Products Directive

**DOI:** 10.1136/tobaccocontrol-2014-051822

**Published:** 2014-08-14

**Authors:** Hélia Costa, Anna B Gilmore, Silvy Peeters, Martin McKee, David Stuckler

**Affiliations:** 1 Department of Sociology, University of Oxford, Oxford, UK; 2 Department for Health, University of Bath and UK Centre for Tobacco and Alcohol Studies, Bath, UK; 3 Department of Public Health and Policy, London School of Hygiene & Tropical Medicine, London, UK

**Keywords:** Tobacco Industry, Public Policy, Tobacco Industry Documents

## Abstract

**Objective:**

The tobacco industry spends large sums lobbying the European Union (EU) institutions, yet whether such lobbying significantly affects tobacco policy is not well understood. We used novel quantitative text mining techniques to evaluate the impact of industry pressure on the contested EU Tobacco Products Directive revision.

**Design:**

Policy positions of 18 stakeholders including the tobacco industry, health NGOs and tobacco retailers were evaluated using their text submissions to EU consultations and impact assessments. Using Wordscores to calculate word frequencies, we developed a scale ranging from 0–tobacco industry to 1–public health organisations, which was then used to track changes in the policy position of the European Commission's 2010 consultation document, its 2012 final proposal and the European Parliament and Council's approved legislation in March 2014.

**Results:**

Several stakeholders’ positions were closer to the tobacco industry than that of health NGOs, including retailers (ω=0.35), trade unions (ω=0.34) and publishers (ω=0.33 and ω=0.40). Over time the European Commission's position shifted towards the tobacco industry from ω=0.52 (95% CI 0.50 to 0.54) to ω=0.40 (95% CI 0.39 to 0.42). This transition reflected an increasing use of words pertaining to business and the economy in the Commission's document. Our findings were robust to alternative methods of scoring policy positions in EU documents.

**Conclusions:**

Using quantitative text mining techniques, we observed that tobacco industry lobbying activity at the EU was associated with significant policy shifts in the EU Tobacco Products Directive legislation towards the tobacco industry's submissions. In the light of the Framework Convention on Tobacco Control, additional governance strategies are needed to prevent undue influence of the tobacco industry on EU policy making.

## Introduction

Starting in 2009, the 2001 European Union (EU) Tobacco Products Directive (TPD), regulating the manufacture, marketing and sale of tobacco products, began a process of revision to reflect recent scientific and market developments in the tobacco sector. Ultimately the revision proposed to balance a stricter regulation for tobacco manufacture, market and sales, mainly for health reasons, with the minimisation of economic costs of such regulation. This led ultimately to agreement on a revised directive—hereinafter referred to as the 2014 TPD—which came into force in May 2014.^1^

From the outset, the process was mired in controversy. The EU's public consultation on the proposed revision received an unprecedented number (over 85 000) of submissions, many of which were later found to be duplicates. In mid-2012, Swedish Match accused the Health Commissioner, John Dalli, of fraud, which eventually led to his resignation in October 2012.^2^ The next day, in a remarkable twist, the Brussels headquarters of several NGOs active in tobacco control were burgled and documents and computers stolen.^3^

It is well-known that the tobacco industry launched a massive campaign against the 2014 TPD, including targeting specific members of the EU's institutions, third party mobilisation and financing of studies to attempt to disprove the need for revision.^4^ In particular, the tobacco industry has been known for emphasising the economic costs of increasing regulation, while downplaying health benefits.^5^ ^6^ A 2012 analysis of leaked internal Philip Morris documents found that its main lobbying strategy was to ‘push’ (amend) or ‘delay’ the TPD revision proposal,^7^ consistent with its attempts to influence the original 2001 TPD.^8^ While its success in delaying the revision is evident from the observation that the timeline slipped by over a year,^9^ ^10^ to our knowledge it is not known whether industry pressure was able to push the legislation's position to favour the tobacco industry. The tobacco industry asserts that public health organisations such as the Smoke Free Partnership actually skewed the EU policy in their favour,^11^ whereas these public health groups claim that tobacco companies succeeded in undermining the legislative process.^12^

To test these competing views, this paper evaluates changes in the main drafts of the 2014 TPD over time using automated content analysis to determine how the policy position of the EU changes with respect to the position of pressure groups.

We first quantify the textual changes that occurred using Wordscores and assessed whether they were more closely associated with the positions of public health organisations or the tobacco industry.^13^ ^14^ Wordscores has been widely applied in political science to code policy positions for party manifestos and lobbyist positions,^15^ ^16^ including European Commission documents.^17^ It scores the policy positions in documents based on the frequency of words. We drew on documents authored by the tobacco industry and public health organisations as a basis for Wordscores to map the positions of tobacco retailers and trade unions, who are known to have played a major role in the tobacco industry's mobilisation tactics,^10^ as well as other actors including associations representing publishers and advertisers which were likely to have occupied more neutral positions. We then test the hypothesis that tobacco industry pressure was able to shift the TPD towards its position from DG-SANCO's (Directorate General for Health and Consumers) initial draft TPD revision document.

## Data and methods

### Sources of data

To identify relevant stakeholders, we drew on the results of a previous review of the TPD revision^10^ to select representatives of the tobacco industry, health NGOs and other stakeholders, including trade unions and publishing, advertising and retail trade associations. We then performed Google searches for publicly available documents in which they expressed their positions in English. These included position papers, extended comments on the Commission's consultation paper and comments on the impact assessment performed by RAND Europe (a non-profit institution providing research services). We extended our search to include documents that were sent to the EU Commission by stakeholders, and retrieved through a series of Freedom-of-Information requests performed in 2013 and 2014.^10^ This yielded a total of 20 documents from 18 stakeholders, written between 2010 and 2013.

**Figure 1:**
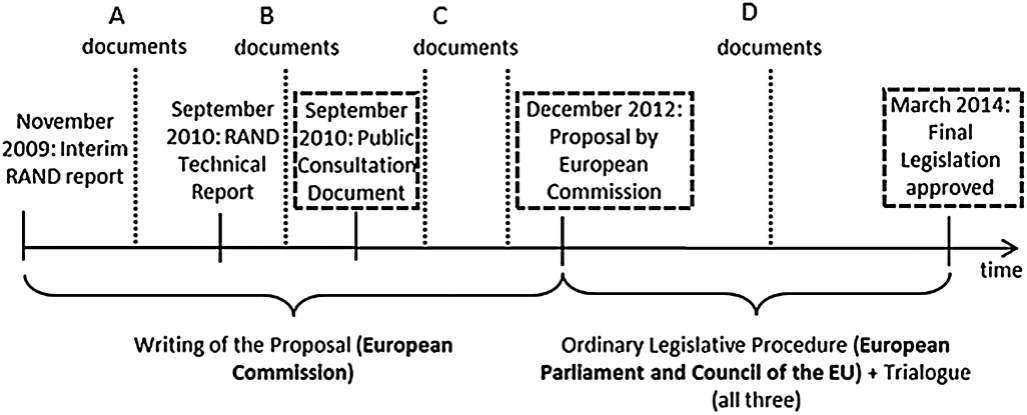
European Union (EU) Tobacco Products Directive Timeline. Documents used to measure the EU's position boxed. Timing of documents used in analysis and set out in table 1 is shown in dotted lines.

Figure 1 summarises the timeline of the TPD process, and table 1 lists the associated stakeholder documents used in the analysis at each juncture. All texts are available on request. The documents identifying the position of stakeholders were collected at different stages of the process. The first two came during the Commission's initial drafting of the proposal between 2009 and 2012. RAND Europe consultants were contracted by DG SANCO to undertake an initial assessment of the impacts of revising the TPD, and consultations from relevant stakeholders were invited by the commission (time points A and B in figure 1). DG-SANCO also held a public consultation, permitting industry to submit its positions alongside those of other stakeholders (time point C). More stakeholder submissions were made when the TPD process reached the European Parliament and Council of Ministers in 2013 (time point D).

**Table 1:** Description of documents used

Entity	Abbreviation	Side	Approximate date	Description and source
Association of European Cancer Leagues and Smoke Free Partnership	ECL/SFP	H	6 June 2012 C	Position paper, online
European Federation of Allergy and Airways Diseases Patients’ Associations	EFA	H	1 July 2013 D	Position paper, online
European Public Health Alliance	EPHA1	H	31 December 2010 C	Opinion on Public Consultation, online
European Public Health Alliance	EPHA2	H	1 May 2013 D	Position paper, online
European Society of Cardiology	ESC	H	1 March 2013 D	Position paper, online
Confederation of European Community Cigarette Manufacturers	CECCM	T	4 March 2010 A	Comment on RAND, FOI
European Cigar Manufacturers Association	ECMA	T	14 December 2010 C	Opinion on Public Consultation, FOI
European Smoking Tobacco Association	ESTA	T	1 March 2010 A	Comment on RAND, FOI
Imperial Tobacco	IT	T	21 February 2013 D	Position paper, online
Japan Tobacco International	JTI	T	16 December 2010 C	Opinion on Public Consultation, FOI
Philip Morris Limited	PML	T	28 February 2013 D	Opinion to UK Health Department, online
Philip Morris International	PMI1	T	15 December 2010 C	Opinion on Public Consultation, online
Philip Morris International	PMI2	T	20 October 2010 B	Comment on RAND, online
Association of German Magazine Publishers	GM	S	1 December 2012 C	Position paper, FOI
Association of Communication Companies, representing the interests of the advertising agencies in Belgium	ACC	S	8 December 2010 C	Opinion on Public Consultation, FOI
European Communities Trade Mark Association	ECTA	S	15 December 2010 C	Position paper, online
European Magazine Media Association	EMMA	S	6 December 2012 C	Position paper, FOI
European Trade Union Confederation (workers)	EFFAT	S	1 December 2010 B	Comment on RAND, FOI
Joint statement between several trade mark associations	TrMark	S	3 July 2013 D	Position paper, online
Retailer Working Group	RWG	S	22 April 2011 C	Position paper, FOI
European Commission	Consultation		1 March 2010	Consultation, online
European Commission	Commission		19 December 2012	Proposal, online
Commission, EU Parliament, Council	Final		14 March 2014	Legislation, online

Health NGO's (H), Tobacco Industry (T) and Other Stakeholders (S).

EU, European Union; FOI, freedom of information request.

These documents include three iterations of the 2014 EU TPD revision used to assess the EU position: the consultation document of September 2010, the final Commission proposal of December 2012, and the final document approved by the European Parliament and the Council in March 2014. These texts were taken from the European Commission and the European Parliament websites.^18^ ^19^

### Measuring policy positions

To compare policy positions taken by stakeholders with the content of the evolving EU legislation we used the scaling algorithm Wordscores.^13^ ^14^ Wordscores infers policy positions, or scores, for new documents—‘virgin texts’—on the basis of documents with known scores, ‘reference texts’. It uses the frequency of words in each document, relative to the total number of words in a text, based on the assumption that agents with different policy positions use different wording which reflects their ideology or stance. For example, the tobacco industry more frequently invokes arguments about the economy and business than public health actors.^5^ When an unknown text includes more text about the economy and business, it is more likely to reflect a tobacco friendly position than one from a health actor.

The relative frequency of a given word w contained in a given reference text r, F_wr_, is used to compute the conditional probability that we are reading text r given that we are reading word w*.* This probability is then used to construct a score, S_w_, for each word w as a weighted average of all the scores of reference texts where word w shows up, weighted by the calculated conditional probability. In a second stage, the calculated word scores are used to compute an overall document score for each virgin text v, ω_v_, as the sum of the scores of words contained in it weighted by their relative frequency F_wv_:1
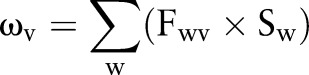


This approach has been previously validated for political texts and economy policy speeches,^15^ ^16^ and has also been applied to European Commission documents.^17^

In the first step of the analysis, we transferred EU documents from pdf to text^20^ and then manually removed superfluous information, including all interest group names and their descriptions, headers and footers, contact details and citations from Commission documents. We created a frequency matrix using the program JFreq in R^21^ which further reduced words to their roots and removed stop words, numbers and symbols and estimated the frequency distribution of words across documents.

In the second part of the analysis, we introduced the scores for the reference texts, which formed a basis for classifying the virgin texts. In view of the known polarisation between the tobacco industry and public health actors, we coded all 8 tobacco industry texts as 0 and the 5 health NGO texts as 1. Text on e-cigarettes was excluded because of the heterogeneity in policy positions held by public health organisations. These reference texts were then used to quantify scores for documents representing the positions of ‘other stakeholders’, including some identified as having been mobilised by and having close associations with tobacco companies, such as tobacco retailers and trade unions^10^ ^12^ ^22^ and others which may have been more neutral, such as organisations representing advertisers and publishers.

In the final step we tracked changes in the EU policies by comparing word scores of the initial EU Commission consultation paper dated 1 September 2010 through to the final proposal dated 19 December 2012. We then analysed the final legislation, voted on by the Parliament in plenary in February 2014 and subsequently approved by the Council on the 14 March 2014.

### Rescaling wordscores

One limitation of Wordscores is that the estimated word scores of virgin texts are not directly comparable to reference texts. Since reference texts tend to have overlapping, non-discriminating words, their word scores tend to be pulled towards the middle of the scale.^23^ To adjust for this limitation, we rescaled the wordscores using the Martin-Vanberg (MV) transformation, developed to facilitate comparability.^24^ We estimated positions for the reference texts along with the virgin texts, and use the most extreme positions observed among the calculated word scores to rescale scores for each virgin text as follows:2
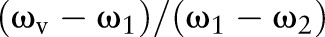


where ω_v_ again stands for the raw score of each virgin text and ω_1_ and ω_2_ for the estimated scores of those virgin texts with the most extreme values. Thus we present both raw and MV transformed word scores, with 95% CIs. All word scores were calculated using STATA V.13.0.

## Results

### Frequencies of word use by different actors

Table 2 shows the word frequency matrix for the most 15 common words used in each document that was part of the analysis. Frequencies are presented as a percentage of the total number of words in each document. The first three texts are the official EU documents and the rest are divided into one of three categories, health NGOs, tobacco industries and other stakeholders. The matrix shows that the frequency of ‘health’ in the health NGO documents, which was a greater concern on the part of NGOs, was about 1.71% of words, corresponding to twice the frequency of health language in tobacco documents, of 0.87%. Over time the word root ‘health’ decreased from 1.50% of total words per document in the initial Commission proposal to 1.21% of total words in the final approved legislation. Similarly, the word root ‘warn’ was twice as frequent among health NGO documents, 1.68%, compared with tobacco industry ones, of 0.69% and declined from 1.57% to 1.18% in official EU documents. The opposite pattern occurred for finance language, such as the root ‘econom’, which albeit not among the 15 most common word roots was the focus of arguments by the tobacco industry.^10^ The average frequency of this word in health documents was 0.05%, while it was 0.14% in the tobacco industry documents. It gradually increased in the EU documents from zero instances in the consultation document to 0.20% in the Commission proposal to 0.25% in the final document, reflecting a greater use of such language than tobacco companies.

**Table 2:** Word frequency matrix, in percentages

	Tobacco	Product	Smoke	Health	State	Consum	Warn	Member	Market	Packag	Cigarett	Commiss	Impact	Direct	European Union
Consultation	5.17	4.79	1.05	1.50	3.07	1.12	1.57	3.00	1.57	1.42	1.27	0.15	0.15	1.35	0.52
Commission	3.78	4.05	0.57	1.29	2.39	0.75	1.24	2.37	0.98	0.82	0.75	1.24	0.04	1.51	0.15
Final	4.04	4.19	0.67	1.21	2.25	0.58	1.18	2.25	0.95	0.90	0.75	1.01	0.03	1.40	0.10
Health															
ESC	0.27	0.33	0.30	0.19	0.07	0.11	0.08	0.07	0.02	0.05	0.04	0.05	0.08	0.05	0.11
EPHA1	3.89	2.88	0.62	2.02	1.63	0.86	2.41	1.09	1.63	1.32	1.09	0.39	0.23	0.70	0.54
EPHA2	4.89	4.49	0.80	1.68	1.04	0.40	1.20	1.12	0.96	1.28	0.64	0.56	0.32	0.72	1.28
ECL/SFP	4.36	3.17	1.09	2.38	0.99	0.30	2.87	0.79	0.69	0.99	1.68	0.10	0.20	0.79	0.20
EFA	2.57	1.97	2.72	2.27	0.15	0.30	1.82	0.15	0.00	1.66	0.61	0.15	0.30	0.30	0.30
Average	3.20	2.57	1.11	1.71	0.78	0.39	1.68	0.64	0.66	1.06	0.81	0.25	0.23	0.51	0.49
Tobacco															
PML	1.11	2.02	0.96	0.98	1.04	0.34	0.41	0.85	1.40	0.39	1.53	2.54	1.32	0.47	1.50
PMI1	2.65	1.72	2.08	0.72	1.04	0.48	0.40	0.68	0.72	0.72	1.72	0.40	0.36	0.44	0.80
PMI2	1.65	0.69	1.25	1.19	0.65	0.37	0.69	0.39	0.96	0.73	0.87	0.47	2.00	0.19	1.03
ECMA	2.33	2.74	0.82	1.22	1.17	1.40	1.69	1.22	0.82	1.40	0.12	0.47	0.06	0.06	0.29
CECCM	1.80	0.36	0.00	0.00	0.90	0.36	0.00	0.54	0.90	0.00	0.00	1.26	3.77	1.26	0.00
IT	1.69	2.51	0.56	1.01	1.13	1.01	0.98	1.13	0.90	0.30	0.75	2.14	0.26	0.83	1.24
JTI	0.42	0.44	3.50	1.12	0.28	2.39	0.87	0.09	0.31	0.55	0.86	0.19	0.45	0.13	0.06
ESTA	6.67	4.17	0.92	0.75	0.79	0.79	0.48	0.61	1.01	0.61	0.75	0.22	0.83	1.05	0.83
Average	2.29	1.83	1.26	0.87	0.88	0.89	0.69	0.69	0.88	0.59	0.83	0.96	1.13	0.55	0.72
Other stakeholders															
EFFAT	4.53	1.46	1.05	0.57	0.00	0.16	0.16	0.00	0.57	0.32	0.81	0.32	0.97	0.32	1.78
ECTA	2.57	2.76	0.00	0.37	0.18	1.10	0.18	0.00	0.18	2.21	0.18	0.18	0.18	0.37	0.92
RWG	2.85	2.39	0.97	0.66	0.56	0.71	0.10	0.41	1.37	0.76	0.66	0.41	0.25	0.15	1.32
EMMA	2.72	2.72	0.00	0.00	0.63	0.63	0.21	0.42	0.00	0.00	0.21	0.21	0.42	1.26	0.63
ACC	2.12	6.35	0.00	0.18	1.06	0.35	0.00	0.00	1.59	3.70	0.00	0.00	0.00	0.88	0.71
TrMark	0.19	1.32	0.00	1.13	0.00	1.32	0.94	1.13	0.38	1.70	0.00	0.38	0.19	0.19	0.38
GM	2.74	5.16	0.32	2.26	0.16	1.45	2.10	0.16	1.13	3.71	0.00	0.00	0.00	0.32	0.00
Average	2.53	3.17	0.33	0.74	0.37	0.82	0.53	0.30	0.75	1.77	0.27	0.21	0.29	0.50	0.82

Frequency matrix created with JFreq; proportion of frequency of each word on total of words calculated manually. Columns organised from most frequently occurring word roots on the left to less frequent on the right, calculated on the totality of the document set.

ECL/SFP, Association of European Cancer Leagues and Smoke Free Partnership; EFA, European Federation of Allergy and Airways Diseases Patients’ Associations; EPHA1, European Public Health Alliance 1; EPHA 2, European Public Health Alliance 2; ESTA, European Smoking PMI, Philip Morris International; PMI1, Philip Morris International 1; PMI2, Philip Morris International 2; ECMA, European Cigar Manufacturers Association; CECCM, Confederation of European Community Cigarette Manufacturers; IT, Imperial Tobacco; JTI, Japan Tobacco Internal; EFFAT, European Trade Union Confederation (workers); RWG, Retailer Working Group; EMMA, European Magazine Media Association; ACC, Association of Communication Companies TrMark, Joint statement between several trade mark associations; GM, Association of German Magazine Publishers.

### Estimating policy positions of differing actors

Based on tobacco industry and public health documents, we next estimated policy positions using Wordscores. Table 3 presents the estimated raw score for each text, its SD, the MV score and associated 95% CIs, as well as the numbers of total and unique scored words.

**Table 3:** Policy position estimations

Texts	Number of unique words	Total words scored	Raw word score (SE)	MV transformed word score	95% CI (MV)	
					Lower bound	Upper bound
Consultation	689	2592	0.40 (0.004)	0.520	0.50	0.54
Commission	1314	11 181	0.38 (0.002)	0.445	0.43	0.46
Final	1235	12 115	0.36 (0.002)	0.404	0.39	0.42
European Magazine Media Association	309	840	0.34 (0.007)	0.33	0.29	0.38
European Trade Union Confederation	674	2210	0.34 (0.005)	0.34	0.31	0.37
Retailer Working Group	942	3965	0.34 (0.003)	0.35	0.33	0.36
Association of Communication Companies	363	1120	0.34 (0.006)	0.35	0.31	0.38
European Communities Trademark Association	356	1019	0.34 (0.006)	0.35	0.32	0.39
Joint Statements of Trade Mark Associations	366	976	0.35 (0.007)	0.37	0.33	0.41
Association of German Magazine Publishers	384	1218	0.36 (0.006)	0.40	0.37	0.43

Raw scores calculated using Wordscores module in Stata. CIs calculated based on MV transformation. Results presented from lowest to highest word scores.

MV, Martin-Vanberg.

As shown in the table 3, the estimated position of groups of other stakeholders is closer to that of the tobacco industry, albeit more moderate. It estimated positions for the European Magazine Media Association of ω=0.33 (95% CI 0.29 to 0.38) and for retailers of ω=0.35 (95% CI 0.33 to 0.36). The European Communities Trade Mark Association was scored at ω=0.35 (95% CI 0.32 to 0.39), and the German Magazine Publishers corresponded to ω=0.40 (95% CI 0.37 to 0.43), relatively closer to public health.

### Estimating textual change in EU documents over time

Finally, we compared how EU legislation evolved throughout the process, relative to the position of the tobacco industry and public health actors.

**Figure 2:**
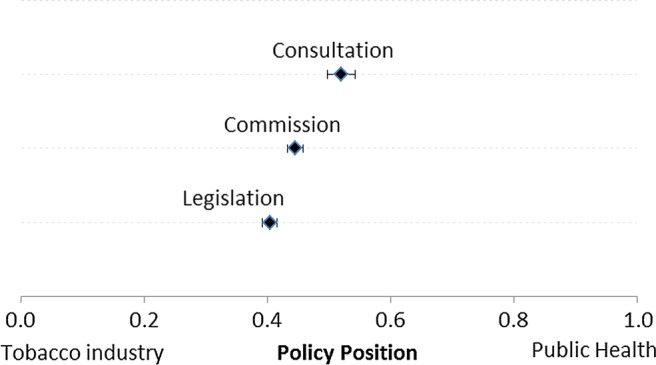
Estimated changes in European Union Policy positions. Word scores estimated in STATA, using tobacco industry and health organisation documents as reference texts. Raw wordscores were rescaled using the Martin-Vanberg transformation (see equation 2). 95% CIs depicted in black bars.

Figure 2 plots the MV scores and the 95% CIs for each of the three EU documents. Consistent with the observation of increasing language about the economy and choice, we observed that the EU's policy position moved towards that of the tobacco industry and mobilised groups, first during the Commission stage from an initial word score of 0.52 (95% CI 0.50 to 0.54) to 0.45 (95% CI 0.43 to 0.48). It further shifted towards the estimated position of the tobacco industry when it reached the EU Parliament and Council, yielding a final word score of 0.40 (95% CI 0.39 to 0.42), which was significantly different from the initial position.

### Robustness tests

To test the robustness of our results to initial classification assumptions, we re-estimated word scores using a different classification method for the texts. We first used as reference texts only the four documents from Philip Morris International and the European Public Health Alliance, to classify the policy positions of the other stakeholders in the tobacco industry and the health NGOs. We then used the estimated positions as reference texts to classify the positions of the other stakeholder groups and the EU documents, as in the initial analysis. As shown in Web appendix 1, none of the results was significantly altered, although the position of mobilised groups began to overlap with that of the tobacco industry for some actors. We further tested alternative methods for rescaling the estimated raw Wordscores, including other commonly used scaling transformations.^13^ None of the results changed qualitatively.

## Conclusion

The revision of the TPD generated a large amount of interest by stakeholders. The tobacco industry, in particular, developed a comprehensive strategy aimed at undermining and delaying the process.^10^ Using quantitative text analysis methods we document that the EU legislation shifted significantly towards the tobacco industry's position and that several other stakeholders, including retailers, were associated with the industry's position.

Our application of automated content analysis has several important assumptions and associated limitations. First, compared with traditional hand-coding methods, automated content analysis provides an objective quantification of policy positions. However, this depends crucially on an assumption that each actor's ideology is expressed through word choice. It is well-established that tobacco industry argumentation often stresses the economic impacts of policies,^5^ which can manifest in its documents’ language. Consistent with possibility, our analysis found that the word root ‘econom’ appeared twice as frequently in tobacco documents as in health NGO documents, whereas health language appeared more frequently in the latter. Further the observation that the frequency of the words ‘health’ and ‘warn’ diminishes, while economic terms increase over time, corroborates our observation of a shift towards the industry's position. Second, quantifying word scores relies on estimating probabilities that are more reliably calculated when reference texts contain large numbers of words that are shared with the documents with unknown positions. We were able to overcome this limitation by using lengthy stakeholder texts from multiple stages of the process. It is also necessary for reference texts to differ from each other, so we included documents from stakeholders known to be diametrically opposed on the TPD. Third, given the complexity of the positions involving e-cigarettes, we excluded submissions relating to them in this analysis. Future research could apply the approach developed in this paper to evaluate the positions of various e-cigarette advocates in relation to public health and tobacco actors. Finally, it is not possible to interpret the raw word scores, but only their relative positions, as the reference texts were used to develop a novel scale ranging from the policy positions of health organisations to those of the tobacco industry. Scores of virgin texts tend to be less extreme than the reference texts, because the virgin texts include more non-discriminating words, which can lead to clustering in the centre of the scale. To facilitate comparability across virgin and reference texts, we followed previous methods to apply the MV transformation.^24^ This rescales scores so that virgin documents can occupy the full range from 0 to 1, which are the values occupied by the two sets of reference documents, rather than bunching in the middle of the scale.

Our results have implications for EU tobacco control policy. While it may have expected a priori that DG-SANCO's initial document reflected a strong public health position, so that the only direction of travel under industry pressure was towards the tobacco industry's position. Nonetheless, according to the Framework Convention on Tobacco Control, policy making should be protected from industry, so evidence suggesting industry influence on EU legislation is of concern.

Our findings of significant textual shifts correspond to substantial policy changes to the TPD.^10^ At the Commission stage, proposals for plain packaging and limitations on point of sale displays were removed. At the Parliament and the Council stage of the process, the size of pictorial health warnings was reduced from 75% to 65% of carton size and the ban on slim cigarettes was rejected. Additionally, the Parliament delayed for 5 years the proposed ban on menthol-flavoured cigarettes, which would have been a major problem for the industry's recruitment of young smokers.

This study's application of automated content analysis, to our knowledge for the first time to tobacco control, has important implications for research and public policy. For researchers, the use of automated content analysis methods to evaluate the association between pressure by lobby groups and public health reforms increases the reliability of the analysis by removing the subjectivity of human coding procedures. It is particularly relevant to quantify the outcomes of pressure in this area due to concerns that powerful industries are able to capture the agenda of public health and effectively water down vital regulation. Future applications of automated content analysis may be useful for detecting potential industry front groups and mobilised third-parties, as Wordscores can be used to identify relative positions of actors. Such an approach would also likely have important applications to analyses of the political economy of alcohol, food and beverage industries, especially where there are large numbers of actors whose policy positions and vested interests may not be well understood.

**What this paper adds**

- The tobacco industry spends large sums lobbying the EU but it is not known whether such lobbying significantly affects policy.
- This paper is the first to apply quantitative text analysis to evaluate the impact of tobacco industry pressure on EU policymaking.
- The analysis demonstrates that industry pressure was associated with a significant shift in contested EU Tobacco Products Directive towards the tobacco industry's position.
- Several stakeholders' positions were found to be closer to the tobacco industry than health NGOs, including retailers, publishers and trade unions.
- The automated content analysis technique could be applied to analyse the political economy of alcohol, food, and beverage industries as well as to better identify tobacco-industry front groups.

## Supplementary Material

Web supplement
