# Supplementary material for: Quantifying the influence of the tobacco industry on EU governance: automated content analysis of the EU Tobacco Products Directive
Source: Tob Control. 2014 Aug 14;23(6):473–8. doi: 10.1136/tobaccocontrol-2014-051822 (PMC4215371; doi:10.1136/tobaccocontrol-2014-051822)
Supplement: Web supplement [file tobaccocontrol-2014-051822-s1.pdf]

Web Appendix 1. Reproduction of Table 3 with alternative classification

Texts	Number of Unique Words	Total Words Scored	Raw Word score (Standard error)	MV Transformed Word Score	95% Confidence Interval (MV)	
					Lower Bound	Upper Bound
Retailer Working Group	942	3965	0.49 (0.0007)	0.39	0.38	0.41
European Trade Union Confederation	674	2210	0.49 (0.0011)	0.39	0.37	0.42
European Magazine Media Association	309	840	0.49 (0.0016)	0.41	0.37	0.45
Association of Communication Companies	363	1120	0.50 (0.0013)	0.45	0.42	0.48
European Communities Trademark Association	356	1019	0.50 (0.0013)	0.48	0.45	0.51
Association of German Magazine Publishers	384	1218	0.50 (0.0012)	0.49	0.46	0.52
Joint Statements of Trade Mark Associations	366	976	0.50 (0.0015)	0.50	0.46	0.54
Final	1235	12115	0.50 (0.0004)	0.52	0.51	0.53
Commission	1314	11181	0.50 (0.0004)	0.55	0.54	0.56
Consultation	689	2592	0.51 (0.0008)	0.62	0.60	0.64

Notes: Raw scores using Wordscores for Stata. Confidence intervals calculated by authors using M-V transformation. Results presented from lowest to highest word scores.
